# PRMT5 inhibition attenuates cartilage degradation by reducing MAPK and NF-κB signaling

**DOI:** 10.1186/s13075-020-02304-x

**Published:** 2020-09-04

**Authors:** Yonghui Dong, Ping Wang, Yongguang Yang, Jincheng Huang, Zhipeng Dai, Wendi Zheng, Zhen Li, Zheng Yao, Hongjun Zhang, Jia Zheng

**Affiliations:** 1 Department of Orthopedics, Henan Provincial People’s Hospital, Zhengzhou University People’s Hospital, Henan University People’s Hospital, No.7, Weiwu Road, Zhengzhou, 450003 Henan Province China; 2 Department of pathophysiology, School of Basic Medical Sciences, Zhengzhou University, Zhengzhou, 450001 China; 3 Department of Molecular Pathology, The Affiliated Cancer Hospital of Zhengzhou University, Henan Cancer Hospital, Zhengzhou, 450008 China

**Keywords:** Osteoarthritis, PRMT5, Chondrocytes, Kinases, DMM model

## Abstract

**Objectives:**

A role for the type II arginine methyltransferase PRMT5 in various human diseases has been identified. In this study, the potential mechanism underlying the involvement of PRMT5 in the pathological process leading to osteoarthritis (OA) was investigated.

**Methods:**

PRMT5 expression in cartilage tissues from patients with OA and control individuals was assessed by immunohistochemical staining. The regulatory and functional roles of PRMT5 in the chondrocytes of patients with OA and control individuals were determined by western blotting and reverse transcription polymerase chain reaction. The effects of the PRMT5 inhibitor EPZ on interleukin-1β-induced inflammation were examined in the chondrocytes of patients with OA and in the destabilized medial meniscus (DMM) of a mouse model of OA.

**Results:**

PRMT5 was specifically upregulated in the cartilage of patients with OA. Moreover, adenovirus-mediated overexpression of PRMT5 in human chondrocytes caused cartilage degeneration. This degeneration was induced by elevated expression levels of matrix-degrading enzymes (matrix metalloproteinase-3 (MMP-3) and matrix metalloproteinase-13 (MMP-13)) in chondrocytes. The activation of the MAPK and nuclear factor κB signaling pathways was evidenced by elevated levels of p-p65, p-p38, and p-JNK. These effects were attenuated by inhibiting the expression of PRMT5. In the mouse model, EPZ inhibited PRMT5 expression, thus protecting mouse cartilage from DMM-induced OA.

**Conclusions:**

Our results demonstrate that PRMT5 is a crucial regulator of OA pathogenesis, implying that EPZ has therapeutic value in the treatment of this cartilage-destroying disease.

## Introduction

Osteoarthritis (OA) is a whole-joint disease characterized by chronic joint pain and loss of function, which occurs in 50% of the population ≥ 65 years of age [1, 2]. The development of OA includes degeneration of the articular cartilage, induction of a synovial inflammatory response, formation of osteophytes, and remodeling of the subchondral bone [3]. Among these pathological changes, cartilage degeneration is the key feature of OA; this is caused by activation of matrix-degrading enzymes, such as matrix metalloproteinase-3 (MMP-3) and matrix metalloproteinase-13 (MMP-13) [4–6]. Both enzymes are regulated by inflammatory cytokines (e.g., tumor necrosis factor α and interleukin (IL)-1β), which are released by chondrocytes [7, 8] and induce the production of MMPs by activating the nuclear factor (NF)-κB and mitogen-activated protein kinase (MAPK) cellular signaling pathways [9, 10]. Necrostatin-1, a cellular catabolic mediator in chondrocytes, also plays a role in OA: it upregulates matrix-degrading enzymes induced by IL-1β [11].

Arginine methylation is a post-translational modification commonly found in mammalian cells and catalyzed by proteins of the arginine methyltransferase (PRMT) family [12, 13]. PRMT5, a type II enzyme, is found in both the cytoplasm and the nucleus of cells [14, 15]; it is involved in many physiological processes, including adipogenesis [16], hematopoiesis [17], and spermatogenesis [18, 19]. The overexpression of PRMT5 in various tumors and in leukemia has also been reported [19–21]. Recent studies indicate that PRMT5 participates in the maintenance of chondrogenic progenitor cells in the limb bud [22]. It is also involved in inflammation, migration of fibroblast-like synoviocytes in rheumatoid arthritis, and induction of an inflammatory reaction in the endothelium [23, 24]. In addition, several studies have shown that PRMT5 regulates the NF-κB pathway [25–28], both via TRAIL [25] and by dimethylation of R30 on the p65 subunit [26].

Here, we report the specific upregulation of PRMT5 in human OA cartilage and show that the inhibition of PRMT5 expression attenuates IL-1β-mediated MMP-3 and MMP-13 expression levels through activation of the MAPK and NF-κB signaling pathways of chondrocytes. These results imply that PRMT5 acts as a catabolic regulator in the pathogenesis of OA.

## Materials and methods

### Clinical sample collection

Clinical specimens (cartilage) were obtained from three patients with OA (two women and one man; age 67.1 ± 8.4 years, range 58–75 years) who underwent total knee arthroplasty. The three patients in the control group (two women and one man; age 30.1 ± 5.6 years, range 18–42 years) underwent total knee arthroplasty related to osteosarcoma involving the knee joint patients; they had no history of OA. All surgeries were performed at the People’s Hospital of Zhengzhou University (Zhengzhou, China). The study was approved by the hospital’s Clinical Research Ethics Committee (approval no. 2017237), and informed consent was obtained from each tissue donor prior to inclusion in this study.

### Animal models

Male C57BL/6 mice (12 weeks old and weighing 20–25 g) used in this study were housed at the Experimental Animal Center of the People’s Hospital of Zhengzhou University and fed a standard diet under specific pathogen-free conditions. An OA model was established in the mice by destabilized medial meniscus (DMM) surgery of the right knee joint, in accordance with a previously described protocol [29]. The 30 mice were randomly divided into three groups of 10 mice each: (1) in the sham group, sham-operated mice were administered vehicle (dimethylsulfoxide); (2) in the DMM group, the mice underwent DMM surgery and were administered vehicle; (3) in the DMM+EPZ group, the mice underwent DMM surgery and were administered the PRMT5 inhibitor EPZ (200 μg/kg). Vehicle or EPZ solution was injected intra-articularly, twice per week for 8 weeks.

Approval for the animal experiments was obtained from the Institutional Animal Care and Use Committee (IACUC) at the People’s Hospital of Zhengzhou University (ethical approval code: 2018526).

### Micro-computed tomography imaging

All mouse (*n* = 10) knee joints were scanned at 100 kV and 98 μA using microcomputed tomography (μCT) (μ-CT50 Scanco Medical, Bassersdorf, Switzerland). The resolution was set to 10.5 μM. Image reconstruction and analysis were performed using the built-in software; these were followed by three-dimensional reconstruction and structural parameter analysis. The structural parameter analysis included determinations of bone volume/tissue volume, trabecular thickness, and trabecular separation.

### Histological and immunochemical analyses

Human cartilage and mouse joint tissue samples were collected and fixed in 4% formalin, then incubated for 3 weeks in 10% EDTA and embedded in paraffin. The tissues were cut into 4-μm coronal sections for hematoxylin and eosin and safranin O-Fast green staining, as well as immunohistochemistry. For cartilage analysis and scoring, safranin O-Fast green staining was performed, according to the OARSI histologic scoring system [29, 30]. Briefly, the extent of the surface damage was scored in a blinded manner at the mouse tibia (lateral tibia (LT) and medial tibia (MT)) as well as femur (lateral femur (LF) and medial femur (MF)); the score for cartilage damage was calculated in a scale of 0 (normal) to 6 (bone loss, remodeling, deformation), based on the OA depth into the cartilage. The OARSI score was calculated based on averaging of maximum score among the four articular surfaces from ten mice.

Immunohistochemistry staining was performed using the DAB tissue staining SP-kit, in accordance with the standard protocol. Tissue sections were incubated overnight with specific anti-MMP-13, MMP-3, PRMT5, p-p38, and p-p65 antibodies at 4 °C and then analyzed by optical microscopy.

### Cell experiments

Cartilage samples collected from patients with OA during total knee arthroplasty were washed, cut into pieces in 4 °C sterile phosphate buffer, and digested with 0.25% trypsin at 37 °C for 30 min; they were then incubated in 0.25% collagenase II at 37 °C for 24 h. The chondrocytes were cultured in DMEM/F12 with 10% fetal bovine serum, 100 U/mL penicillin, and 100 mg/mL streptomycin at 37 °C under 5% CO_2_. Chondrocytes were passaged twice before use in experiments. To study the induction of inflammation, passage 3 chondrocytes were seeded on six-well plates (2 × 10^5^ cells/well) and treated with 10 ng/mL IL-1β for 72 h (Sigma-Aldrich, St. Louis, MO, USA). Levels of PRMT5, MMP-3, MMP-13, Collagen II, Sox9, and ADAMTs5 protein were then determined. To investigate the mechanism by which PRMT5 damages articular chondrocytes, we examined the expression of p38, JNK, ERK1/2, p65, IκB, p-JNK, p-p38, p-ERK1/2, p-IκB, and p-p65 in chondrocytes that had been stimulated with IL-1β for 0, 15, and 30 min.

### Micromass cultures

Micromass cultures were performed as previously described [31]. Briefly, chondrocytes were plated at a density of 2.5 × 10^5^ cells/10-μL drop. The cell culture medium was replaced with fresh culture medium every other day. On days 5 and 7, cartilage differentiation was determined by alcian blue staining; the cartilage was then extracted using 6 M guanidine hydrochloride. The absorbance of the supernatant at 600 nm was measured using a multi-mode microplate reader (BioTek, Winooski, VT, USA).

### Adenovirus vector infection

Human PRMT5 cDNA was cloned and inserted into a pHBAd-MCMV-GFP expression vector (Wuhan Miaoling Biotechnology Co. Ltd., Hubei, China). Empty vectors were used as controls. Human chondrocytes were cultured in DMEM/F-12 medium without fetal bovine serum in 12-well plates. When the cultures had reached 70% confluency, they were transduced with pHBAd-MCMV-GFP-PRMT5 (ad-PRMT5) and the vector control (ad-NC), in accordance with the manufacturer’s instructions. After the direct addition of recombinant adenoviruses to the medium, the transduced cells were incubated for 4 h; the medium was then changed to vector-free medium. Samples were collected for the measurement of protein and mRNA expression, after the virus vector infection 72 h.

### RNA extraction and quantitative real-time PCR

Total RNA was extracted from human chondrocytes using TRIzol reagent (Invitrogen, Carlsbad, CA, USA), in accordance with the manufacturer’s instructions. cDNA of each sample was synthesized from 1 μg of total RNA using the Revert Aid First Strand cDNA Synthesis Kit (Thermo Scientific, Waltham, MA, USA). The mRNA expression levels of *PRMT5*, *MMP-3*, *MMP-13*, *Collagen II*, *Sox9*, and *ADAMTs5* were measured by using the qRT-PCR method, and the relative primers are listed in Table S1. Target mRNA expression levels were normalized against GAPDH. The relative expression levels were computed using the 2^−ΔΔCt^ method.

### Western blotting

The total protein of cartilage samples from OA patients was extracted using tissue homogenizer, and then RIPA buffer supplemented with protease and phosphatase inhibitor (Boster BIO, Wuhan, China) was added and ultimately the cell lysates were centrifuged at 16000×*g* for 20 min to remove the tissue. Protein concentrations of the samples were determined using a BCA protein assay kit (Boster BIO, Wuhan, China). Proteins were extracted from cells using 100 μL of RIPA buffer supplemented with protease and phosphatase inhibitor. Lysates were centrifuged for 20 min at 10,000*g*; 20 μg of total cellular protein per sample was loaded on a 10% sodium dodecyl sulfate-polyacrylamide gel, in accordance with the manufacturer’s protocol (Boster Bio, Wuhan, China). Protein samples were then transferred to the PVDF membranes (Millipore, Billerica, MA, USA) and blocked for 1 h with 5% skim milk in Tris-buffered saline with 0.1% Tween-20. The blots were then probed overnight at 4 °C with rabbit primary antibodies against PRMT5, MMP-3, MMP-13, Collagen II, Sox9, ADAMTs5, p38, JNK, ERK1/2, p65, IκB, p-JNK, p-p38, p-ERK1/2, p-IκB, p-p65, and GAPDH. After the blots were washed three times with Tris-buffered saline with 0.1% Tween-20, they were incubated with anti-mouse or anti-rabbit secondary antibodies. Immunoreactivity was detected with enhanced chemiluminescence (Bio-Rad Laboratories, Munich, Germany).

### Statistical analysis

The data are presented as means ± standard deviations of at least three independent experiments. Student’s *t* tests were used to analyze the differences between the two groups, and one-way analysis of variance with the SNK post hoc test was used to compare the differences among the three groups. A two-tailed *p* value of < 0.05 was considered to indicate statistical significance.

## Results

### PRMT5 is upregulated in OA chondrocytes

**Fig. 1:**
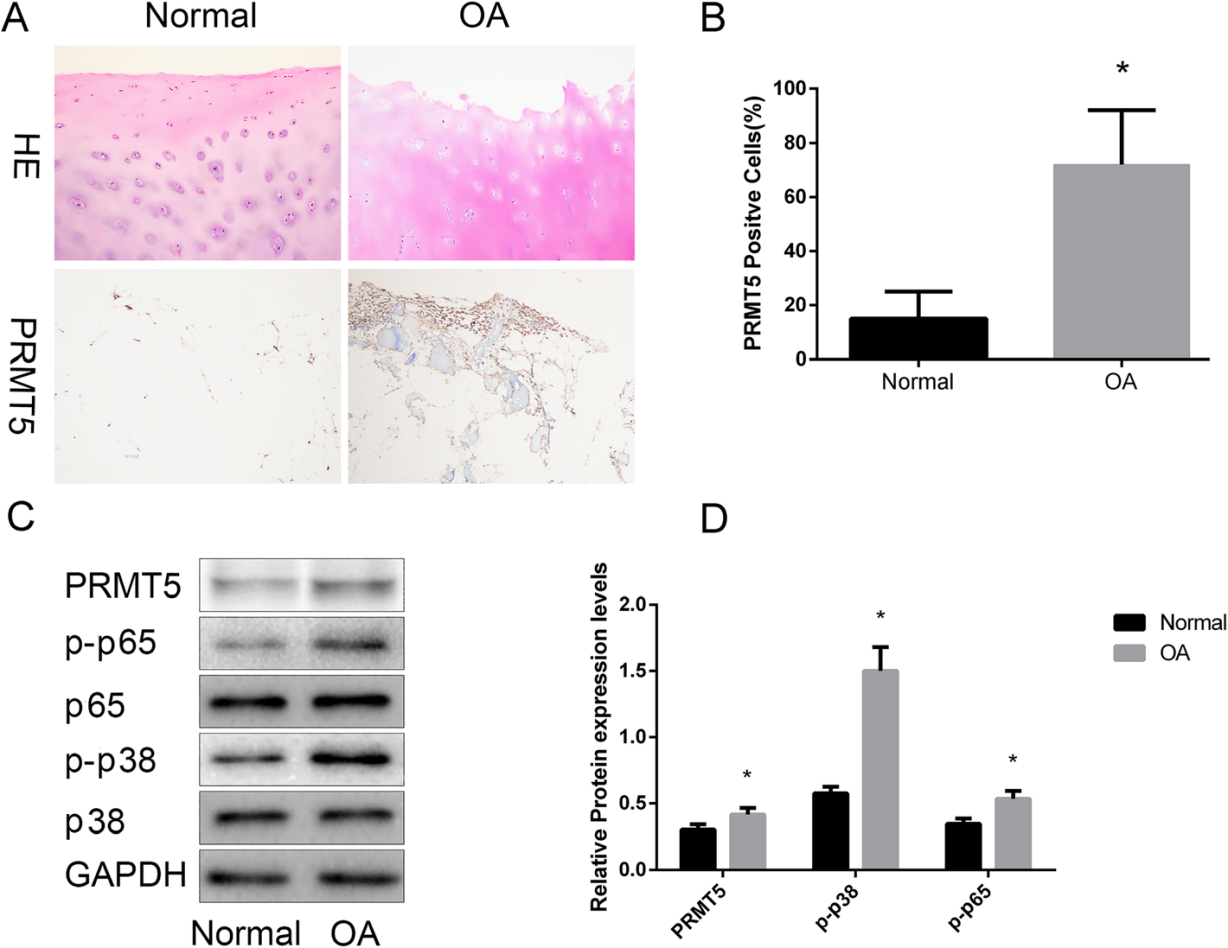
Overexpression of PRMT5 in human osteoarthritis (OA) cartilage. **a** Hematoxylin and eosin staining and representative immunohistochemical staining for PRMT5 in human cartilage in the control and OA groups. Scale bar = 100 μm. **b** Increase in the numbers of PRMT5-positive cells in human OA cartilage vs. control cartilage. Results are presented as percentages. **c** Representative western blots show increases in the expression levels of PRMT5, p-p65, and p-p38 in OA tissues; levels of p65, p38, and GAPDH were identical to those in the control group. **d** Histogram showing the relative protein expression levels of PRMT5, p-p65, and p-p38 in cartilage tissue. Data are presented as mean ± SD from 3 samples for each group in 3 independent experiments. Data were analyzed using Student’s *t* test. *P < 0.05 vs. the normal group

To study the role of PRMTT5 in the pathogenesis of OA, we measured the expression of PRMTT5 in the cartilage from OA patients or normal individuals using immunohistochemistry. The results showed that PRMT5 protein levels were markedly elevated in OA human cartilage, compared with the control group cartilage (Fig. 1a, b). Furthermore, western blotting showed upregulation of PRMT5, p-p38, and p-p65 expressions (Fig. 1c), which indicated the activation of NF-κB and MAPK subtypes in OA human cartilage.

### PRMT5 overexpression causes cartilage degeneration

**Fig. 2:**
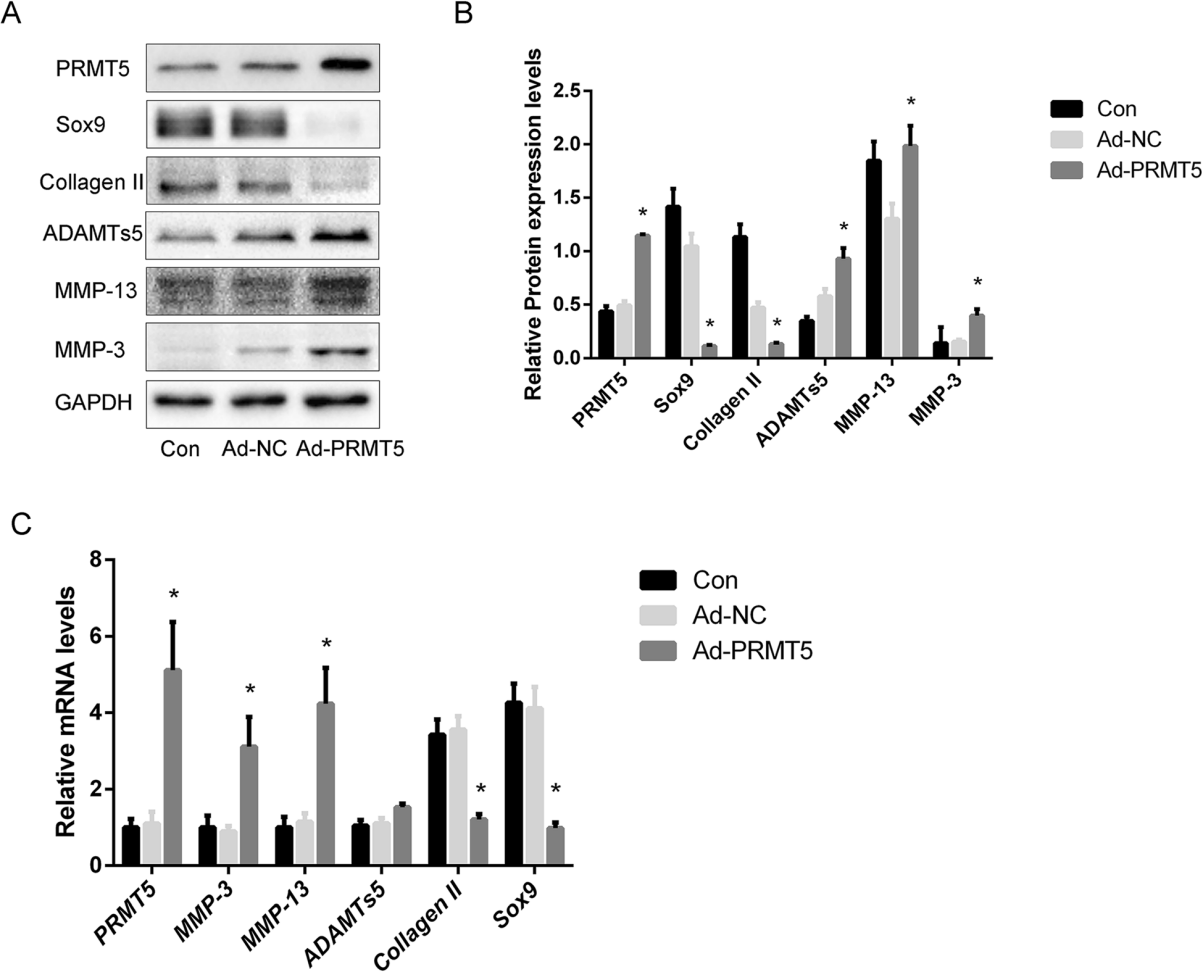
Overexpression of PRMT5 contributes to cartilage degeneration. **a** Expression levels of MMP-3, MMP-13, ADAMTs5, Sox9, Collagen II, and PRMT5 in human chondrocytes from the control, Ad-NC, and Ad-PRMT5 groups were determined by western blotting. **b** Histogram showing the relative protein expression levels of PRMT5, MMP-3, MMP-13, ADAMTs5, Sox9, and Collagen II in human chondrocytes. **c** Changes in *MMP-3*, *MMP-13*, *PRMT5*, *ADAMTs5*, *Sox9*, and *Collagen II* gene expression levels in human chondrocytes transduced with Ad-PRMT5. Data of independent experiments (*n* = 3) were presented as mean ± SD and analyzed using the one-way analysis of variance with the SNK post hoc test. *P < 0.05 vs. the Ad-NC group

The role of PRMT5 in the regulation of cartilage metabolism was investigated by the transduction of primary culture human chondrocytes with a PRMT5 adenovirus vector, which resulted in the overexpression of the protein. As shown in Fig. 2a, compared with the control group, PRMT5 overexpression induced the upregulation of MMP-3, MMP-13, and ADAMTs5, as well as the downregulation of Collagen II and Sox9. Elevated PRMT5 levels also caused the upregulation of *MMP-3*, *MMP-13*, and *ADAMTs5* mRNA expression, whereas the downregulation of *Collagen II* and *Sox9* mRNA expression was observed (Fig. 2c).

### PRMT5 inhibition attenuates IL-1β-stimulated induced chondrocyte catabolism

**Fig. 3:**
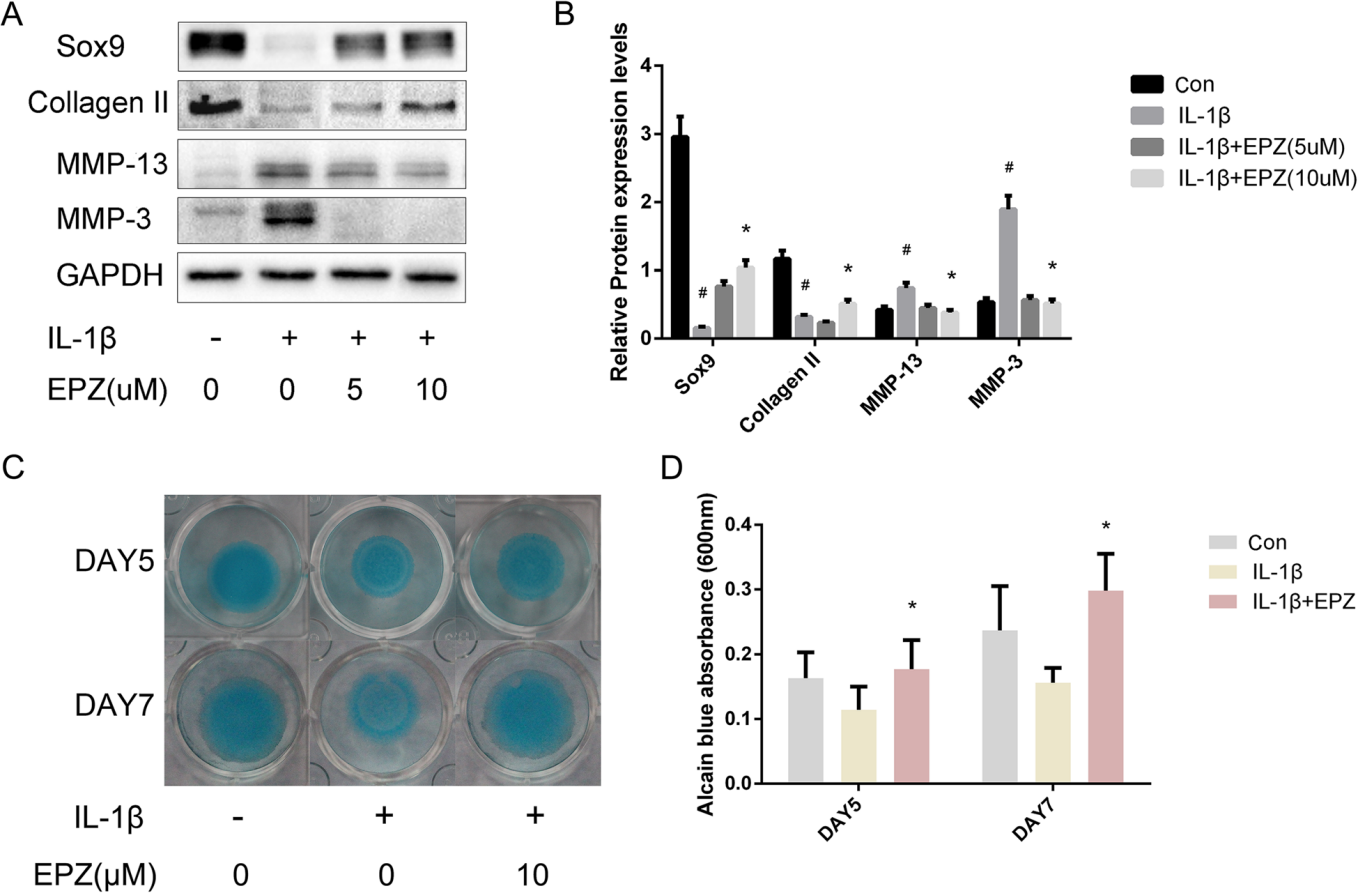
Inhibition of PRMT5 attenuates IL-1β-stimulated chondrocyte catabolism. **a** Representative western blots showing the expression levels of MMP-3, MMP-13, Sox9, and Collagen II in IL-1β-stimulated human chondrocytes in response to EPZ. **b** Histogram showing the relative protein expression levels of MMP-3, MMP-13, Sox9, and Collagen II in human chondrocytes. **c** Alcian blue staining of micromass cultures incubated in the absence of IL-1β, with or without EPZ. **d** Alcian blue-stained cultures were extracted using 6 M guanidine hydrochloride; the absorbance of the supernatant was measured at 600 nm. Data of independent experiments (*n* = 3) were presented as mean ± SD and analyzed using the one-way analysis of variance with the SNK post hoc test. *P < 0.05 vs. the IL-1β group. ^#^*P* < 0.05 vs. the con group

To determine the effect of PRMT5 inhibition on IL-1β-stimulated chondrocyte degeneration, primary cultures of human chondrocytes were co-treated with EPZ in the presence or absence of IL-1β for 72 h. As shown in Fig. 3a, MMP-3 and MMP-13 protein expression were upregulated, while Collagen II and Sox9 protein expression were downregulated in IL-1β-treated cells; in cells treated with both IL-1β and EPZ, the IL-1β-mediated responses were attenuated. The ability of EPZ to affect early chondrogenesis by primary culture human chondrocytes stimulated with IL-1β for 5 and 7 days was determined by using in vitro high-density micromass cultures. Figure 3c, d also shows that IL-1β reduced the alcian blue incorporation, an effect counteracted by the presence of EPZ.

### PRMT5 inhibition confers protection against experimental OA

**Fig. 4:**
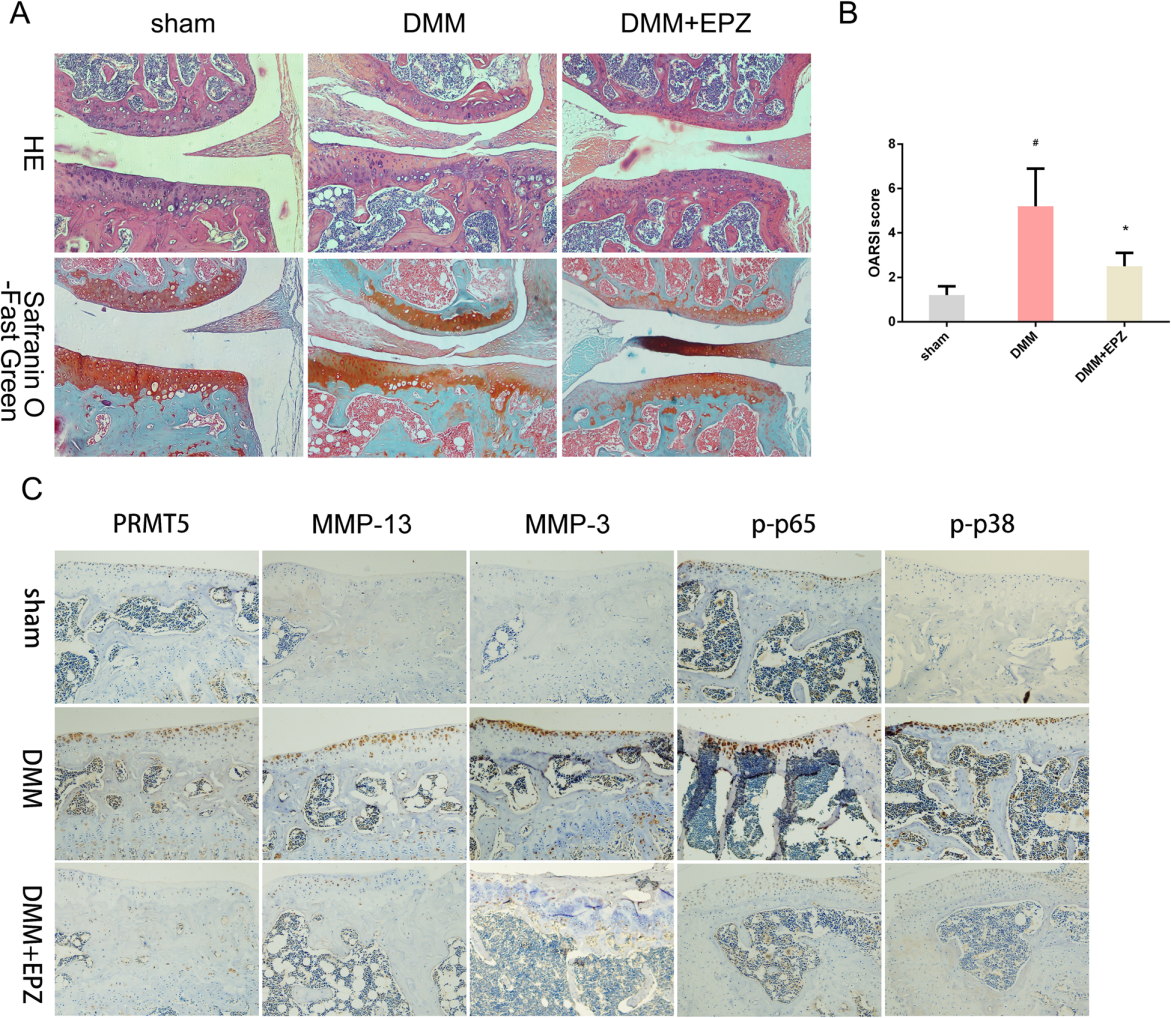
EPZ reduces the progression of experimental OA. **a** Hematoxylin and eosin and safranin O-fast green staining of the coronal sections from the knee joint cartilage of sham, DMM, and DMM+EPZ mice. Scale bar = 100 μm. **b** Histopathological examination using the Osteoarthritis Research Society International criteria to assess cartilage degeneration. **c** MMP-3, MMP-13, PRMT5, p-p38, and p-p65 expression, as detected by immunohistochemical staining. Data were presented as mean ± SD from 10 samples. Data were analyzed using the one-way analysis of variance with the SNK post hoc test. *P < 0.05 vs. the DMM group. ^#^*P* < 0.05 vs. the sham group

**Fig. 5:**
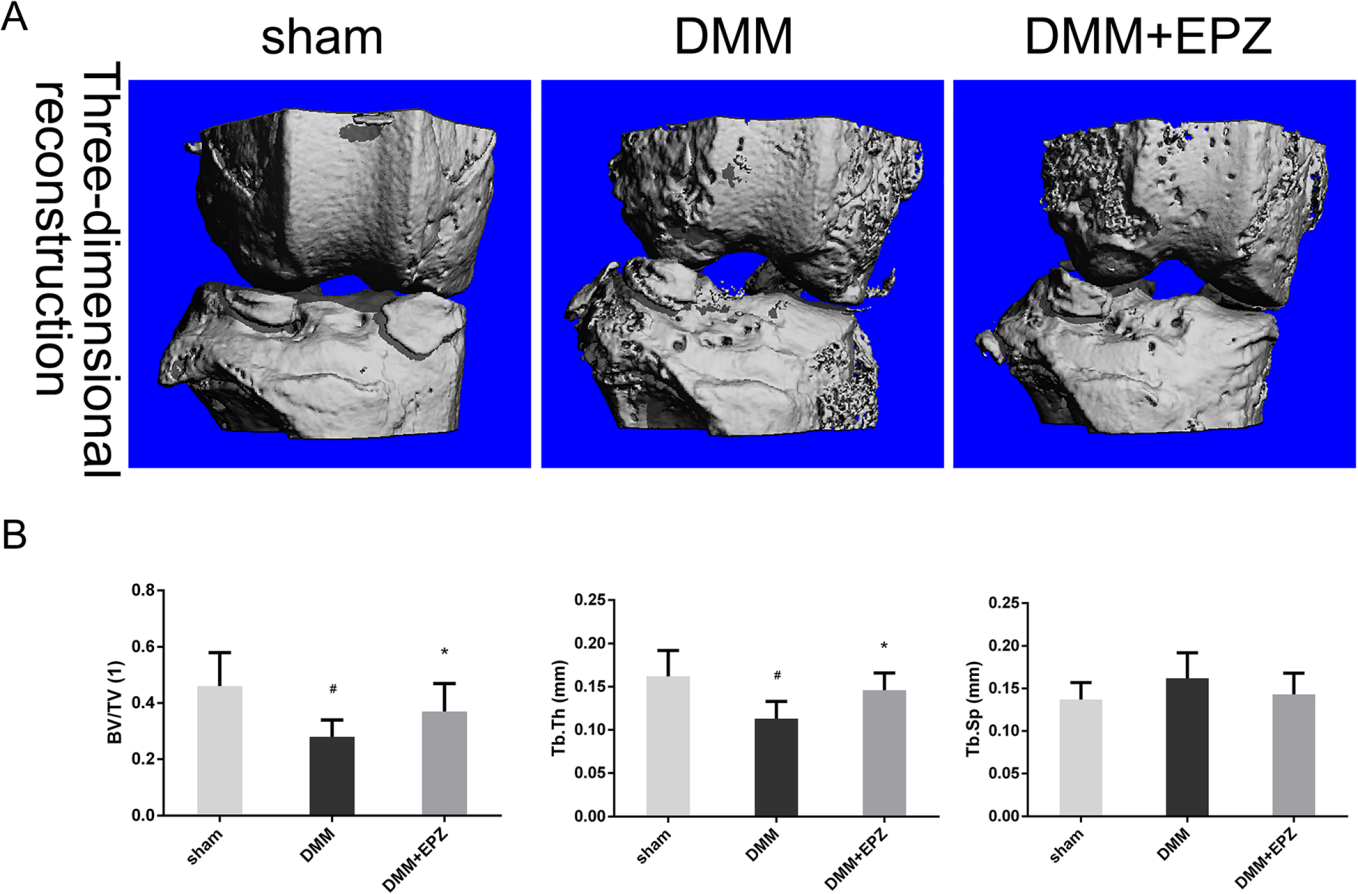
EPZ inhibits DMM-induced subchondral bone loss in mouse tibia. **a** Three-dimensional images of the knee joints in each group, reconstructed using the built-in μCT software. **b** Structural parameters of the tibial subchondral bone, analyzed according to trabecular bone volume/tissue volume (BV/TV), trabecular thickness (Tb. Th), and trabecular separation (Tb. Sp). Data are presented as mean ± SD from 10 samples. Data were analyzed using the one-way analysis of variance with the SNK post hoc test. *P < 0.05 vs. the DMM group. ^#^*P* < 0.05 vs. the sham group

The effect of PRMT5 inhibition on experimental OA pathogenesis was assessed in a DMM mouse model. DMM results in cartilage erosion, osteophyte formation, and subchondral bone plate thickening [32, 33]; therefore, it serves as a model of OA. Figure 4a shows that EPZ significantly inhibited cartilage destruction in DMM mice; it also reduced the DMM-induced upregulation of MMP-3, MMP-13, and PRMT5 expression, whereas the downregulation of p-p38 and p-p65 expression were observed in the DMM+EPZ group, implying that EPZ can neutralize the activation of the NF-κB and MAPK pathways induced by DMM (Fig. 4c). The Osteoarthritis Research Society International score indicated a protective effect of EPZ on the articular cartilage (Fig. 4a–c). Dramatic changes in total subchondral bone tissue were revealed by μCT, with 47.5% and 36.2% reductions in bone volume/tissue volume and trabecular thickness, respectively, in DMM mice compared to sham-operated mice. Furthermore, trabecular separation increased by 16.8% in DMM mice, whereas EPZ attenuated tibial subchondral bone loss, compared to vehicle-treated DMM mice (Fig. 5).

### NF-κB and MAPK mediate PRMT5 activity in chondrocytes

**Fig. 6:**
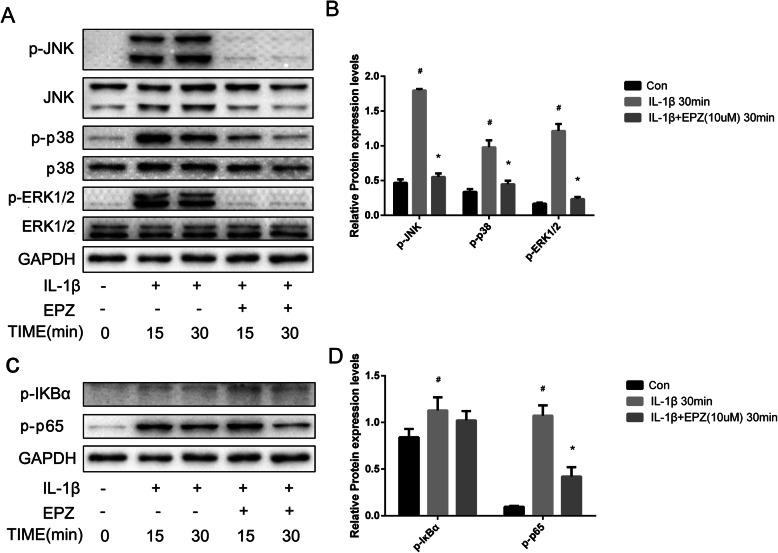
EPZ inhibits IL-1β-induced NF-κB and MAPKs in human chondrocytes. **a** Human chondrocytes were cultured for 16 h in serum-free medium, pretreated with EPZ (10 μM) for 24 h, then stimulated with IL-1β (10 ng/mL) at the indicated times. GAPDH, p38, JNK, ERK1/2, p-JNK, p-p38, and p-ERK1/2 expression levels were analyzed by western blotting. **b** Histogram showing the relative protein expression levels of p-JNK, p-p38, and p-ERK1/2 in human chondrocyte. **c** Chondrocytes were treated as above; the expression levels of GAPDH, p65, IκB, p-IκB, and p-p65 were analyzed by western blotting. **d** Histogram showing the relative protein expression levels of p-IκB, and p-p65 in human chondrocyte. Data of independent experiments (*n* = 3) were presented as mean ± SD and analyzed using the one-way analysis of variance with the SNK post hoc test. *P < 0.05 vs. the IL-1β group. ^#^*P* < 0.05 vs. the con group

To investigate the mechanism by which PRMT5 damages articular chondrocytes, we examined the activation statuses of the NF-κB and MAPK pathways in chondrocytes that had been stimulated with IL-1β in the presence or absence of EPZ. Figure 6 shows that EPZ inhibited the upregulation of phosphorylated-IκBα, p65, p38, and JNK, which had been induced by IL-1β. Thus, the effects of EPZ on IL-1β-induced PRMT5 included inhibition of the NF-κB and MAPK signaling pathways.

## Discussion

There remains no effective treatment for OA, and the mechanisms underlying its pathogenesis are poorly understood [34]. PRMT5 is abundantly expressed in a wide variety of human cells [35], and its expression is associated with the occurrence and progression of various diseases [36]; however, its role in the development and progression of OA has been unclear. Our study showed that PRMT5 contributes to OA pathogenesis in mice by modulating the expression levels of MMP-3 and MMP-13 in chondrocytes; in addition, PRMT5 is upregulated in IL-1β-stimulated chondrocytes of patients with OA. The ability of PRMT5 to increase the levels of catabolic factors suggests that it is a crucial catabolic regulator involved in OA.

Increasing evidences suggest that OA is a disease of the whole joint including the cartilage, synovium, ligaments, bone, and bone marrow. In recent years, the subchondral bone has been considered to be one of the main pathological features of OA [37–39]. In OA, there is a significant loss of trabecular bone from the tibial subchondral bone. Together with bone marrow lesions, this bone loss is closely linked to pain and is predictive of the severity of cartilage damage in OA. Osteoclasts play a role in the remodeling of the subchondral bone. In this study, we found that EPZ can inhibit DMM-induced subchondral bone loss. These study results were consistent with a previous study which showed that subchondral bone loss is in the DMM OA model [40]. On the contrary, some studies had found that trabecular bone volume/tissue volume (BV/TV) and tibial thickness increased in the subchondral bone of the DMM model [41, 42]. PRMT5 has been demonstrated to regulate osteoclast differentiation and protect against the bone-related effects of ovariectomy in our previous study [43]. However, this study revealed that EPZ by intra-articular injection could not affect the activation of osteoclast in the subchondral bone (Fig. S1). Evidences suggest that cross-talk between chondrocytes and subchondral bone may be involved in the pathology of OA [44, 45]. Therefore, we conferred that EPZ that mainly affected the metabolism of chondrocytes involved in the development of OA may be plausibility. NF-κB comprises a family of ubiquitously expressed transcription factors involved in immunity, stress responses (including responses to mechanical stress), and inflammatory diseases [46]. Our results indicated that the upregulation of MMP-3 and MMP-13 by IL-1β-induced PRMT5 during cartilage destruction is mediated by NF-κB. However, because the effect was only partially blocked by EPZ, other PRMT5-inducing signaling mechanisms may also be involved.

Ramachandran et al. showed that chondrocytes without PRMT5 were unable to undergo hypertrophic differentiation [47], a process related to articular cartilage degeneration. Dongying Chen et al. demonstrated the important roles of PRMT5 in inflammatory responses, cell proliferation, and migration and invasion of fibroblast-like synoviocytes in rheumatoid arthritis; notably, they showed that these responses are mediated by the NF-κB and AKT pathways [24]. Taken together, these results implicate PRMT5 in synovial lesions of OA. In contrast, Sun and colleagues showed that PRMT5 promotes the expression of Sox9 to maintain type collagen II expression levels in human juvenile costal chondrocytes [48]. The discrepancies among studies may be related to the different cell lines used in the respective experiments.

A limitation of our study was that only one time point was examined in the DMM mouse experiment, which evaluated the effect of PRMT5 on OA progression. In addition, while our results implicate the NF-κB pathway in PRMT5-mediated OA pathology, the specific mechanism linking the molecular events to cartilage damage remains unknown. Future research should involve analysis of multiple time points in animal studies and a larger number of patients in clinical studies to better understand the specific downstream mechanisms of PRMT5 in the development of OA.

## Conclusions

Our study identified PRMT5 as a newly described catabolic regulator of OA pathogenesis. Overexpression of PRMT5 upregulated the expression levels of matrix-degrading enzymes in chondrocytes of patients with OA, an effect that could be partially attributed to the activation of the NF-κB and MAPK signaling pathways. Although the specific mechanism is not yet known, our results suggest that PRMT5 should be further explored for the prevention and treatment of OA.

## Supplementary information

**Additional file 1: Table S1.**

**Additional file 2: Figure S1.**

**Additional file 3: Figure S2.**

**Additional file 4: Figure S3.**

## Data Availability

The datasets used and/or analyzed during the current study are available from the corresponding author on reasonable request.

## Abbreviations

DMM: Destabilized medial meniscus
EPZ: EPZ015666
IL: Interleukin
MAPK: Mitogen-activated protein kinase
MMP: Matrix metalloproteinase
NF-κB: Nuclear factor kappa-B
OA: Osteoarthritis
PRMT5: Protein arginine *N*-methyltransferase 5
μCT: Microcomputed tomography
