# Supplementary material for: PRMT5 inhibition attenuates cartilage degradation by reducing MAPK and NF-κB signaling
Source: Arthritis Res Ther. 2020 Sep 4;22:201. doi: 10.1186/s13075-020-02304-x (PMC7650297; doi:10.1186/s13075-020-02304-x)
Supplement: Supplementary file 1 — Additional file 1: Table S1. [file 13075_2020_2304_MOESM1_ESM.docx]

| \| Gene \| \| --- \| | Primer sequence (5′–3′) |
| --- | --- | --- |
| *Sox9*  AGCGAACGCACATCAAGAC  CTGTAGGCGATCTGTTGGGG  *Collagen II*  TGGACGCCATGAAGGTTTTCT  TGGGAGCCAGATTGTCATCTC  *ADAMTs5*  GAACATCGACCAACTCTACTCCG  CAATGCCCACCGAACCATCT  *MMP-3* AGTCTTCCAATCCTACTGTTGCT  TCCCCGTCACCTCCAATCC  *MMP-13*  ACTGAGAGGCTCCGAGAAATG  GAACCCCGCATCTTGGCTT  *PRMT5*  CTGTCTTCCATCCGCGTTTCA  GCAGTAGGTCTGATCGTGTCTG  *GAPDH*  CCATGTTCGTCATGGGTGTGAACCA  GCCAGTAGAGGCAGGGATGATGTTC | |

Primer sequence
