# Supplementary figures and images for: PRMT5 inhibition attenuates cartilage degradation by reducing MAPK and NF-κB signaling
Source: Arthritis Res Ther. 2020 Sep 4;22:201. doi: 10.1186/s13075-020-02304-x (PMC7650297; doi:10.1186/s13075-020-02304-x)

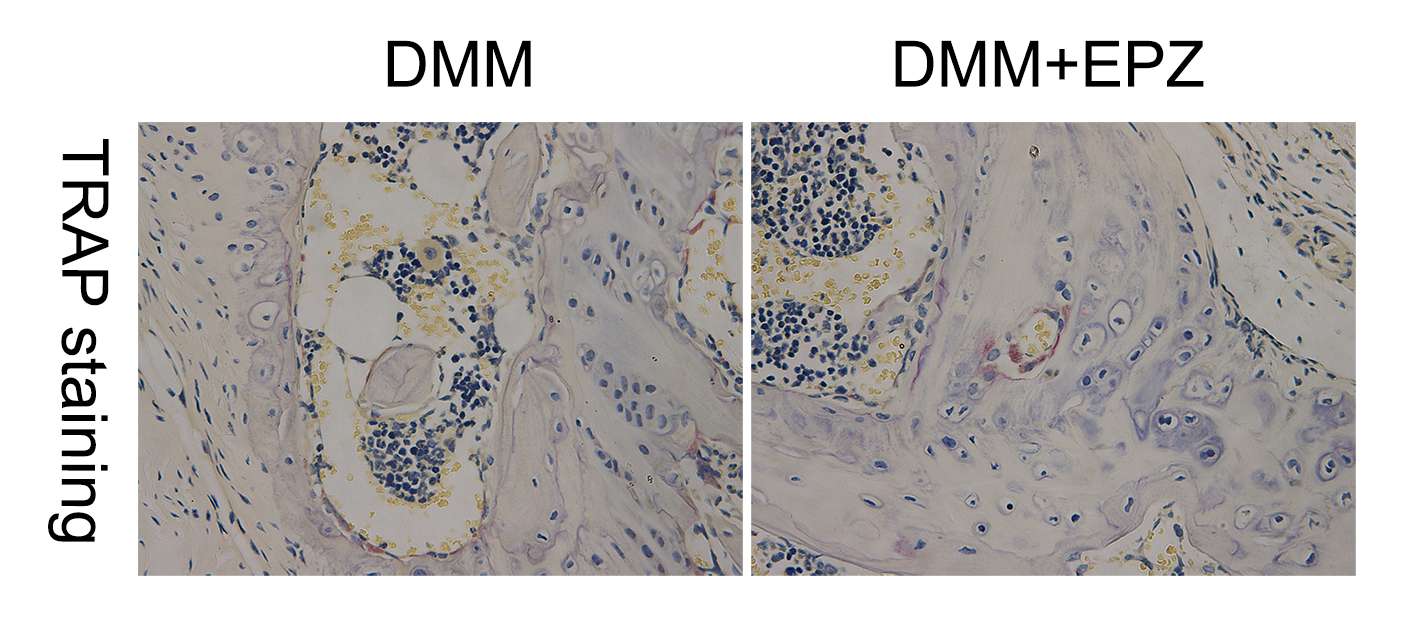

Supplement: Supplementary file 2 — Additional file 2: Figure S1. [file 13075_2020_2304_MOESM2_ESM.tif]

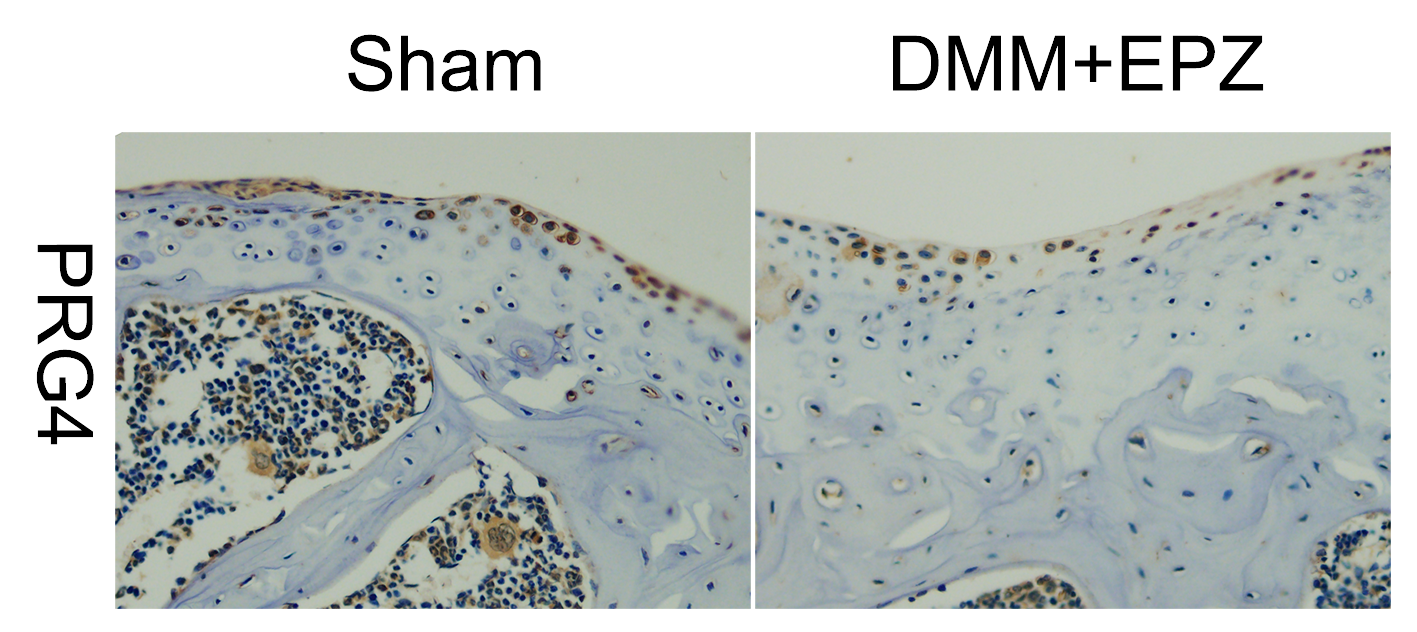

Supplement: Supplementary file 3 — Additional file 3: Figure S2. [file 13075_2020_2304_MOESM3_ESM.tif]

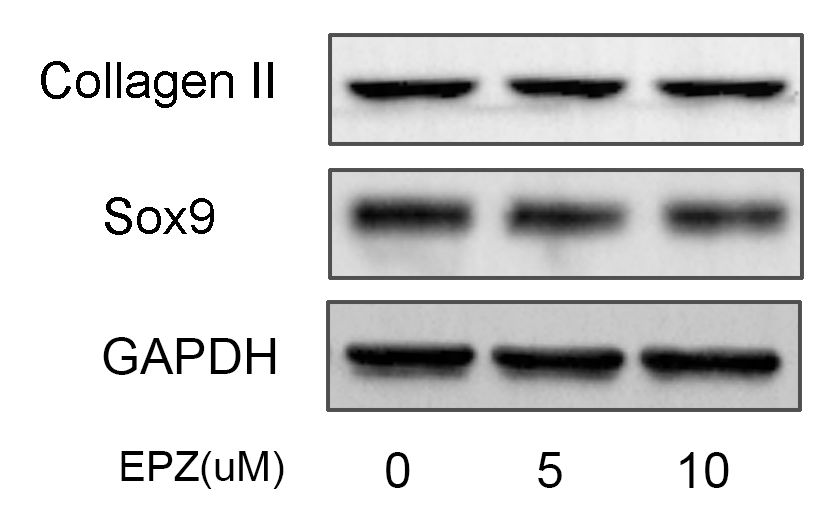

Supplement: Supplementary file 4 — Additional file 4: Figure S3. [file 13075_2020_2304_MOESM4_ESM.tif]
